# Every-Day Ailments

**Published:** 1887-04-02

**Authors:** 


### Till the Doctor Comes.

[Inquiries and Communications for this column should be addressed, "Physician," care of the Editor of The Hospital.]

### XIV.—EVERY-DAY AILMENTS.

*Stroke.*—Caused by arrest of circulation through some portion of the vessels of the brain. Patient usually falls down insensible, and there is found to be paralysis of one side of the body. Lift him into bed, keep the head well raised, and apply cold rags to his head. Be careful about giving him anything, as he may not be able to swallow. Send for a doctor.

*Foreign Bodies in Eye.*—If under lower lid, easily removed by drawing down the lid, and removing with the corner of a handkerchief or towel. If under the upper lid, may sometimes be removed by rubbing it towards the inner corner of the eye, or by pulling the upper eyelid forward over the lower; if these means fail, place a knitting needle or pencil across the upper part of the lid with one hand, take hold of the eyelashes with the other, and turn the lid inside out over the knitting needle, which must at the same time be pressed downwards a little. By these means all the under surface of the upper lid may be thoroughly examined. Sometimes a sharp fragment of steel or dust will strike the clear part of the eye itself, and remain embedded there. It is very difficult for an unskilled person even to see these small particles, as they can only be detected in a certain light; so if there is any suspicion of such an accident, application must be made to a doctor. They have to be lifted out bodily by the point of a sharp penknife.

*Stye.*—A small boil on the eyelid. Best brought forward as quickly as possible by hot poulticing and fomentations. Directly a yellow point forms on its apex, it should be pricked with a lancet or fine needle, when the matter will escape, and there will be at once great relief from pain.

*Cold in the Eye.*—May be best treated with poppy fomentations applied externally. Unless very severe, bathing with alum lotion (a teaspoonful to a pint of water), and letting a little of the lotion run into the eye, is preferable. If it gets worse, or does not speedily recover, application should at once be made to a medical man; and all eye cases require great care, for though to the ordinary observer the diseases may appear similar, yet they may be very different, and require very opposite treatment.

*Chilblains.*—An inflammation of a portion of skin, caused by cold, and generally occurring on the hands and feet. Best treated by frictions with stimulating liniments; of these, compound camphor liniment is one of the best. Many people find great relief from painting chilblains with strong tincture of iodine. When they become what is called broken, they must not be rubbed, but dressed with vaseline and cotton wool.

*Chapped Hands.*—Protect them from cold cutting winds, and dry them carefully after washing. A lotion of equal parts of glycerine, spirits of wine, and rose water is very serviceable, and does not make the hands smart so much as glycerine alone. If they are very bad, spermaceti ointment will cause less smarting. These applications should be used freely at night, and old gloves should then be put on till the morning.

*Earache.*—Apply a hot bran poultice, or laudanum fomentations. If these do not relieve the pain, mix one drop of laudanum with two or three drops of sweet oil in a teaspoon; warm them well over a candle, and drop into the ear, then re-apply the hot dressings. In cases of habitual discharge from the ear, always consult a doctor.

*Ingrowing Toe Nail.*—Caused by wearing tight boots, or by cutting down the corner of the nail too much. If only just commencing, may be cured by continuously packing oiled cotton wool under the outer edge of the nail, so as to raise it, and push back the skin that tends to overhang. This side of the nail also may be gradually scraped down with glass till it is so thin that it yields. More severe cases must go to the medical man.

*Boils and Abscesses.*—Must in almost all cases be poulticed, or treated by hot fomentations. These relieve the pain more than anything. Sometimes when the skin is irritable, a crop of small boils will appear around the part from the irritation caused by the poultice. A piece of lint, with a hole cut in the centre, should then be thickly spread with zinc ointment, and this should be applied first, with the hole over the centre of the abscess, and the poultice over that.

*Warts.*—Best treated by touching them with nitric acid repeatedly, at intervals of about a week. Stick caustic leaves a nasty black stain, and is less efficacious. The nitric acid is best applied by dipping a small slip of wood into it (the end of a match answers capitally), and then touching the wart; by this means too much acid is not used at one time.

*Corns.*—Remove as far as possible all pressure and friction by protection with unirritating plaster, and easy boots. Keep them cut down assiduously, and in time they may disappear. Iodine paint is often useful. The remedies are legion, but effective ones are still wanting.

*Whitlow.*—A very severe inflammation of the fingers or thumbs. Use hot poultices and fomentations. The matter in these cases is generally deeply seated, and the skin is remarkably thick, so if left to itself, it will burrow for a long distance—often into the palm of the hand—when it may cause wide-spread and permanent mischief. These cases, therefore, should always be opened early, and be under the treatment of a medical man.

(*To be continued.*)

